# A Scoping Review of Game-Based Learning for Metacognitive Learning in Primary and Junior Middle Schools

**DOI:** 10.3390/bs16060979

**Published:** 2026-06-12

**Authors:** Juan Li, Huanghui Zhu, Yanxiong Xiang, Lingyun Huang

**Affiliations:** Department of Curriculum and Instruction, The Education University of Hong Kong, Hong Kong; s1166373@s.eduhk.hk (J.L.); zhuhuanghui559@gmail.com (H.Z.); xiangyx@eduhk.hk (Y.X.)

**Keywords:** game-based learning, metacognition, self-regulation, primary and middle school, scoping review

## Abstract

Game-based learning (GBL) has gained widespread attention as an innovative pedagogical approach, yet its potential to enhance students’ metacognitive learning remains underexplored. Guided by self-regulated learning (SRL) theory, the review investigates how GBL design features, such as goal-setting, real-time feedback, progress visualization, and reflection tools, scaffold students’ planning, monitoring, and evaluation strategies. A systematic search across Web of Science, Scopus, and ProQuest identified the studies, which included data from physical classrooms, online learning environments, and mixed settings. This scoping review synthesizes evidence from 11 peer-reviewed studies conducted between 2015 and 2025 to evaluate the impact of GBL on metacognitive learning in primary and junior middle school contexts. Findings reveal that GBL effectively supports metacognitive learning through real-time feedback and progress indicators, though planning and evaluation scaffolds are less comprehensively addressed. Furthermore, digital trace data and behavioral logs are emerging as robust tools for assessing metacognitive processes, offering deeper insights than self-reports alone. However, the review identifies critical gaps, including insufficient focus on junior middle school students, limited representation of non-STEM disciplines, and uneven theoretical grounding across studies. The findings underscore the need for theory-driven design and balanced scaffolding to maximize GBL’s potential in fostering metacognitive competence. This study also provides practical insights for educators to foster students’ metacognitive learning by effectively integrating games into educational practices.

## 1. Introduction

Game-based learning (GBL) has emerged as a transformative pedagogical approach, integrating game mechanics and dynamics into educational settings to enhance learner engagement and facilitate the acquisition of complex knowledge and skills (Lin et al., 2020; Thompson & Gillern, 2020; Yu et al., 2020). This domain encompasses a broad spectrum of applications, including serious games designed explicitly for educational purposes (Krath et al., 2021; Wang & Huang, 2021), and gamified systems that overlay elements such as points and badges onto traditional learning contexts (Kim & Castelli, 2021; Meng et al., 2024). Unlike purely entertainment-focused media, the defining characteristic of GBL is the structured fusion of gameplay with explicit learning objectives (Alaswad & Nadolny, 2015; Shi & Shih, 2015). While the motivational benefits of GBL are well documented, recent research has increasingly focused on understanding how GBL facilitates students’ metacognitive abilities that are critical for learning in these environments.

The theory of self-regulated learning (SRL) provides a critical opportunity to understand students’ metacognitive abilities in learning contexts. According to SRL theory, metacognitive abilities refer to students’ ability to plan, monitor, and evaluate their cognitive processes (Winne & Hadwin, 1998). SRL theory conceptualizes learning as a cyclical process. As modeled by Zimmerman (2002), in the forethought phase, students prepare for learning tasks by setting objectives and selecting strategies. Then, they shift focus to self-monitoring and strategy use during the performance phase and then evaluate performance, reflect on outcomes, and adjust future strategies based on successes or challenges in the self-reflection phase. Every phase of the SRL cycle requires students to utilize metacognitive abilities to control the learning processes and the quality of learning products (Winne & Hadwin, 1998). For example, students use metacognitive skills to ensure their goals are appropriate and achievable. While learning, students constantly monitor and evaluate their learning outcomes and adjust actions based on real-time internal feedback. Finally, students reflect on outcomes, evaluate successes or failures, and refine strategies for future tasks.

Metacognitive learning exhibits distinct developmental trajectories across age groups, and the primary and junior middle school years represent a critical window for their foundational formation. Children aged 7–12 show steady improvements in metacognitive abilities, including planning, monitoring, and control skills (Van Loon & Roebers, 2024; Okada, 2021). Research also finds that a marked decline in metacognitive competencies often occurs during the transition from primary to secondary school (Katsantonis, 2024; Mok et al., 2007), driven largely by a decline in intrinsic motivation and mastery goals rather than by a loss of cognitive capacity (Bouffard et al., 1998). This developmental pattern highlights the urgency of supporting metacognitive growth during early and middle childhood (Leutwyler, 2009), before motivational and contextual disruptions impede further development. However, traditional classroom practices often struggle to engage students in active metacognitive activities (Van Loon & Roebers, 2024; Zepeda et al., 2019). Therefore, GBL, with its interactive and iterative nature, offers a promising avenue to address this challenge by embedding metacognitive practice within motivating, student-centered contexts (Amzalag et al., 2024; Checa-Romero & Giménez-Lozano, 2025). These learning strategies are supported by theoretically sound game design features such as explicit goal-setting prompts, progress visualization, in-game logs, and performance summaries, which stimulate learners to engage in metacognitive planning, monitoring, and evaluation strategies (Amara et al., 2024; Gurbuz & Celik, 2022; Luo, 2021).

Although existing findings are encouraging, the current literature remains fragmented (Braad et al., 2020; Ricker & Richert, 2021; Stephanou & Karamountzos, 2020). While prior reviews have separately investigated game-based learning and metacognition, no review has specifically focused on the intersection of GBL and metacognitive learning strategies within primary and junior middle school contexts. Therefore, this scoping review aims to synthesize and analyze current evidence regarding the influence of GBL on students’ metacognitive learning, specifically among primary and junior middle school students. As justified previously, primary and junior middle school students represent a crucial stage for developing metacognitive skills, laying a foundation for our motivation in this study. Second, GBL is particularly engaging for this age group, aligning with their developmental needs. Additionally, limited research exists on GBL’s impact on metacognitive learning in this population, making them an ideal focus for addressing this gap. The review aims to systematically explore the core connections between GBL and metacognitive learning by identifying the specific types of design features integrated into GBL that can facilitate learners’ metacognitive planning, monitoring, and evaluation. To address these objectives, the following research questions (RQs) have been formulated to guide the review process:

- RQ1: What are the theoretical foundations for the design of GBL in the selected studies?
- RQ2: What are the design features of the selected studies?
- RQ3: What is the impact of GBL features on students’ metacognitive learning in the selected studies?

By systematically reviewing effective game design features and valid assessment approaches, this review offers clear guidance for teachers and game designers seeking interventions that cultivate deep and transferable metacognitive competence, and recommends directions for future empirical research on how digital games may promote broader academic growth.

The following sections present the theoretical background, methodology employing the PRISMA workflow, and results with discussions addressing the research questions.

## 2. Theoretical Background

### 2.1. The Framework of Metacognitive Learning

Flavell’s (1979) important research first defined metacognition as “knowledge and cognition about cognitive phenomena”. He clearly distinguished between two key parts: metacognitive knowledge and metacognitive regulation (Livingston, 2003). Metacognitive knowledge is what learners know about their own abilities, the requirements of different tasks, and which strategies work best in specific situations (Schraw, 1998; Tuononen et al., 2022). This knowledge includes declarative (knowing what), procedural (knowing how), and conditional (knowing when and why) components, which help learners plan, monitor, and evaluate their learning processes (Kassimi, 2025; Teng, 2020). Metacognitive regulation is the active process of controlling one’s thinking to reach learning goals. It enables learners to adjust their mental effort and strategies to optimize performance across different tasks, with evidence showing that these regulation skills can transfer even between different types of learning strategies (Wirth et al., 2025). Research shows that metacognitive learning is critical, as it empowers students to be self-regulatory in their learning and therefore enhances academic performance (Burin et al., 2020; Teng, 2020).

### 2.2. The Mechanism of Game-Based Learning for Metacognitive Learning in Education

Game-based learning (GBL) is defined as immersive learning environments where core game mechanics (e.g., tasks, feedback, progression) and narratives are intentionally integrated with explicit learning goals, including serious educational games (e.g., Minecraft: Education Edition) and digital game-based learning platforms. GBL is different from gamification. GBL refers to full games with embedded learning goals, such as Minecraft: Education Edition (Kucher, 2021; Plass et al., 2015; Sun et al., 2023), while gamification involves superficial game elements like points or badges in non-game contexts. Marc Prensky’s “digital native” hypothesis sparked significant interest in GBL by suggesting that learners born into the digital era naturally prefer interactive, game-like experiences (Prensky, 2001). However, contemporary research refines this view, emphasizing that effective GBL depends more on intentional instructional design than on generational traits alone (Eck, 2006).

The capacity of GBL to foster metacognition is grounded in two key educational theories, Self-Determination Theory (SDT) and Flow Theory, which explain the mechanisms by which GBL promotes metacognition. First, Self-Determination Theory (SDT) contends that GBL’s focus on autonomous choice, competence support, and social connection meets learners’ core psychological needs, including autonomy, competence, and relatedness (Gupta & Goyal, 2022; Proulx et al., 2017; Ryan et al., 2006). This alignment enhances intrinsic motivation to engage in proactive metacognitive behaviors, such as voluntary planning and reflective evaluation (Braad et al., 2020; Checa-Romero & Giménez-Lozano, 2025). Notably, this motivational enhancement may also indirectly promote non-metacognitive outcomes, such as emotional and behavioral engagement (Greipl et al., 2021; Abdul Jabbar & Felicia, 2015; Ninaus et al., 2019), but these outcomes serve as secondary correlates rather than the core focus of this review. Moreover, Flow Theory suggests that game-based learning (GBL) fosters flow states by balancing task difficulty with learners’ competence and using immersive narratives, thereby boosting engagement and motivation (Admiraal et al., 2011; Chang et al., 2017; Csikszentmihalyi, 1990). Empirical studies show that GBL leads to significantly greater flow experiences compared to non-game learning, with learners demonstrating higher concentration, interest, and control over their learning (Chang et al., 2017; Y. Chen et al., 2025). Flow states in GBL are linked to improved learning outcomes, as flow enhances intrinsic motivation and satisfaction, which mediate the relationship between engagement and achievement (Cai et al., 2025). Key game design elements that promote flow include clear goals, adaptive challenges, immediate feedback, autonomy, and sensory immersion, all of which help maintain the balance between task challenge and individual skill (R. Li et al., 2019; Liao et al., 2025).

Existing research indicates that GBL effectively fosters metacognition when its design aligns with the three core metacognitive strategies. In terms of planning, GBL environments use clear mission instructions, planning tools like maps or checklists, and tutorials that prompt learners to consider strategies before starting, which helps reduce cognitive load and supports strategic decision-making (Braad et al., 2020; Goslen et al., 2024). For monitoring, GBL provides progress displays such as progress bars, real-time feedback to identify mistakes early, and resource indicators, such as remaining time, to adjust pace (Geden et al., 2021; Jornevald et al., 2025). These elements allow learners to track their performance and adjust their approach without interrupting gameplay (Geden et al., 2021; Jornevald et al., 2025). For evaluation, GBL uses post-level summaries with key data, outcome screens that show the effect of choices, and replay tools that let learners review their gameplay (Leonardou et al., 2020). Collectively, these design elements support metacognitive learning within an engaging and motivating context (Cloude et al., 2025; Leonardou et al., 2020; Villareale et al., 2020).

## 3. Methodology

**Figure 1:**
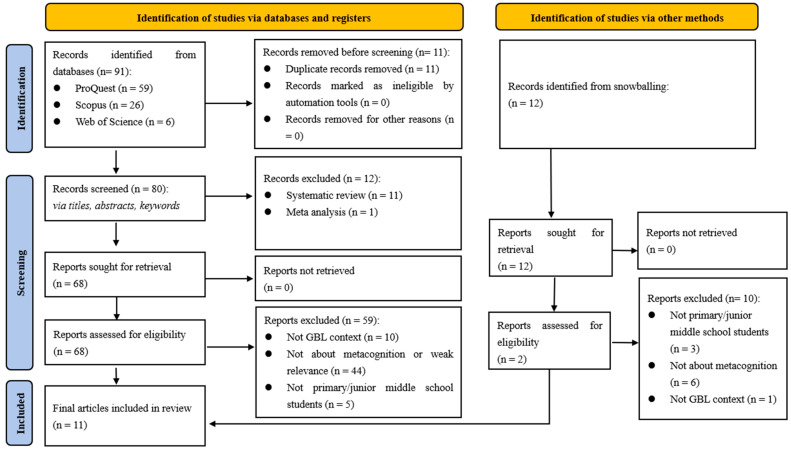
PRISMA flow diagram.

This scoping review was conducted and reported in strict compliance with the relevant requirements of the Report Specification for Systematic Review and Meta-analysis (PRISMA 2020), as proposed by Page et al. (2021). To ensure the transparency and repeatability of the research process, we conducted the research according to its 27-item checklist and used a flow chart to illustrate the three stages of research selection (see Figure 1). The identification and screening of related articles were completed under clearly defined inclusion and exclusion criteria.

### 3.1. Identification

This study selected three core academic databases for literature search: Web of Science, Scopus, and ProQuest. These databases contain a wide range of high-quality, peer-reviewed educational literature that can effectively support the literature base of this review. All searches were completed in one week to ensure the consistency and timeliness of the search results.

We formulated the search strategy for each database based on three fundamental keywords: game-based learning, metacognition, and primary and junior secondary education. Synonyms and alternate spellings of the concepts were included in the search strategy, and Boolean operators were used to combine them (“OR” within groups and “AND” between groups). Table 1 presents the specific search terms. When searching, we applied the criteria to include the literature. First, the study should be written in English. Second, it should be a journal article published between 2015 and 2025 in a peer-reviewed academic journal. Third, full papers should be accessible for download. The database search was limited to the title, abstract, and keyword fields to enhance relevance and avoid a large number of irrelevant results that may be introduced by full-text field retrieval. Consequently, 91 articles were identified from the three databases.

### 3.2. Screening

The screening process was divided into two stages. First, we imported the identified articles into Zotero (version 7.0.32), a reference management system, to detect duplicates, which left 80. Next, we screened the titles and abstracts of the 80 articles against the inclusion and exclusion criteria (Table 2) and removed 71 that were illegible. Finally, nine articles were retained from the three databases. To expand retrieval coverage, we also adopted the snowballing strategy. By checking the reference lists of the above nine qualified documents, 12 potential related studies were identified. After reading the full text, it was confirmed that two of them met all the inclusion criteria. Therefore, 11 studies (nine from the database search and two from snowballing) were finally retained. Appendix A lists the titles, authors, publication years, and journal names of these 11 studies. To facilitate citation and statistical analysis, we assigned a unique study ID to each study.

### 3.3. Coding Process

We adopt a structured coding process to extract and synthesize data from the included literature systematically. Referring to the two-stage method proposed by Webster and Watson (2002), the author-centered analysis was carried out first, and then the standardized data extraction table was developed to align with each research question (See Table 3). For example, to address RQ1, the types of games used in GBL (i.e., serious games and gamified systems) are analyzed. The analysis further identifies specific design mechanics related to metacognitive strategies, including planning (e.g., goal-setting and task description), monitoring (e.g., real-time feedback and progress bars), and evaluation (e.g., performance analysis and explanatory feedback on errors).

To ensure inter-coder reliability, an iterative coding and revision process was adopted. Firstly, the author developed a preliminary coding scheme based on a relevant literature review. Then, the three authors independently coded the same subset of three selected studies to calibrate the application of the scheme. By comparing and discussing the results, the coding scheme was preliminarily optimized. After calibration, the remaining studies were divided equally and coded independently by the three authors. Upon completion, each author’s coding results were reviewed by the other two in a paired-review process. All discrepancies identified during these reviews were resolved through collective discussion until a consensus was reached. This process of independent coding, paired review, and consensus discussion promoted the continuous iterative optimization of the coding scheme. The scheme had been repeatedly adjusted to improve its clarity and accuracy until a stable final version was established and applied to all studies.

## 4. Results

### 4.1. Description of the Selelcted Papers

#### 4.1.1. Publication Information

**Figure 2:**
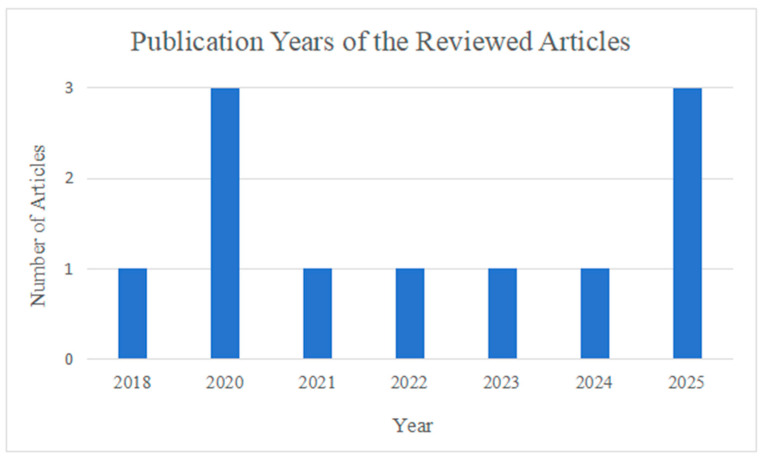
Publication years of the included papers.

**Figure 3:**
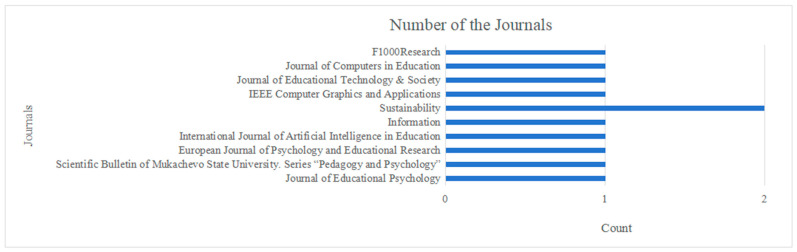
Journals where the selected studies were published.

**Figure 4:**
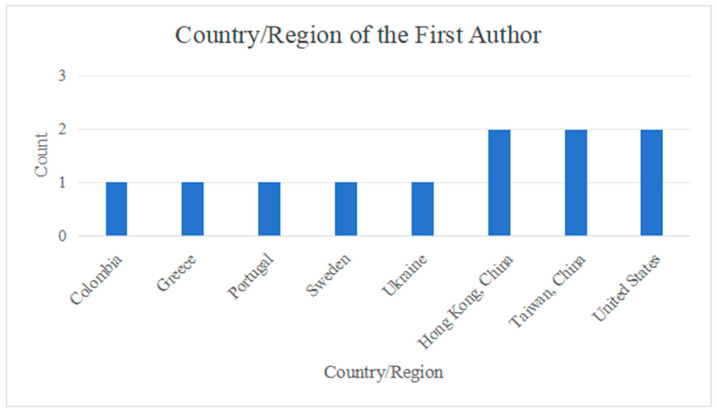
Countries/regions where the first authors are from.

Figure 2, Figure 3 and Figure 4 present the publication information, including the publication year (Figure 2), the published journals (Figure 3), and the countries/regions of the first authors (Figure 4). As shown in Figure 1, the distribution of publication years indicates that most papers were published between 2018 and 2025, with a peak in publication activity in 2020 and 2025. However, this description did not suggest a significant trend on this topic due to a small sample size.

The results in Figure 3 show that the selected papers are distributed across various journals, with two papers from the field of educational psychology, and “Sustainability” having the highest count (2 papers). All other journals, including “F1000Research,” “Journal of Computers in Education,” and others, have only one paper each. This indicates a broader distribution of publications with a slight focus on “Sustainability.”

Figure 4 shows the distribution of publications by country/region. The United States, Taiwan (China), and Hong Kong (China) each contributed two publications, while Ukraine, Sweden, Portugal, Greece, and Colombia each contributed one publication. However, these raw counts should be interpreted with caution as they do not account for differences in population size, research infrastructure, or total scientific output across countries. Direct comparisons between regions with vastly different research capacities (e.g., the United States versus Portugal) may not reflect the relative research intensity in this field.

#### 4.1.2. Sample Sizes of the Included Papers

The reviewed studies varied considerably in sample size, ranging from 6 to 363 participants (M = 141, SD = 116). As shown in Table 4, five studies had sample sizes ranging from 50 to 200 participants. Two studies had larger samples of more than 200 participants, while two studies had smaller samples of fewer than 50 participants.

#### 4.1.3. Educational Levels of the Participating Students

Most of the studies (n = 9, accounting for 81.8%) are targeted at primary school students, as shown in Table 5. Only two studies targeted students at junior middle schools. The age range of the primary school students was 6–10 years and that of the middle school students was 13–15 years old.

#### 4.1.4. Subject Domains

The subject areas involved in the included research can be summarized into three categories (Table 6). STEM (Science, Technology, Engineering, and Mathematics) is the dominant category, with six studies. Studies on language learning account for 36.4% of the total. There is one study focusing on special education.

#### 4.1.5. Research Settings

Table 7 shows the research setting of the studies included in this review. Eight studies were conducted in physical classrooms, and two in an online environment. In addition, one study employed a mixed setting, combining both physical and online environments. No study was conducted in laboratories.

#### 4.1.6. Research Methods

An analysis of the research methods employed across the studies shows a distinct methodological distribution, as summarized in Table 8. Five articles employ quantitative designs. The mixed method design occupies the same proportion. By integrating quantitative data (such as test scores) with qualitative data (such as interviews and reflection texts), the research provides a more comprehensive demonstration of metacognitive processes in the GBL environment. Notably, only one study used a qualitative design to explore perspectives through interviews. This distribution indicates a strong preference for empirical, data-driven approaches, complemented by qualitative insights.

### 4.2. RQ1: Theoretical Foundations for the Design of GBL

As Table 9 illustrates, the theoretical frameworks employed across the reviewed studies reveal a clear dominance of models related to metacognitive learning. Key proponents commonly referenced theoretical frameworks include Zimmerman, Pintrich, and Winne & Hadwin. Other theoretical perspectives are also applied. For example, Self-Determination Theory is used in #5. One study (#9) adopted an integrated framework that combines situational learning theory, an SRL model, and distributed practice principles. It is worth noting that some studies (#2, 3, 7, 8, and 10) did not clearly state their theoretical frameworks.

### 4.3. RQ2: Methodological Features

#### 4.3.1. Game Design Types Integrated in GBL for Metacognitive Learning

Among the 11 studies included in the review, five studies (#1, 2, 3, 4, 7, 9) used serious games to create the GBL to promote metacognitive learning. Six (#5, 6, 8, 10, 11) studies utilized gamification systems (Table 10). According to the design intention, games are divided into traditional games, focused on entertainment, and serious games, with both entertainment and clear educational goals. Serious games have a complete narrative structure, scene design, and clear game goals, and they are systematically integrated into the realistic educational goal in the complete game experience (Krath et al., 2021). By contrast, a gamification system is not a complete game form but a design that embeds individual game elements (such as points and leaderboards) in non-game contexts to enhance participation (Meng et al., 2024). In this study, the gamified systems incorporated elements highly aligned with game-based learning principles. More specifically, there are 12 game types/genres such as adventure/role-playing games (RPG), puzzle-solving games, simulation games, and strategy games (Benson, 2014). Adventure/RPG usually incorporates puzzle-solving tasks that require thinking in a coherent narrative background. Simulations are designed to train users’ skills in specific situations or equipment operations (such as management training and combat simulation). Strategy games are mostly based around conflict situations such as World War II (Benson, 2014).

As shown in Table 11, the most common game types included in the literature are adventure/RPG (#1, 4, and 9), where students assume a specific character (such as a scientist or a store manager) and accomplish task-based goals under the narrative framework, thus making the problem-solving process situational. Simulation games also occupy an important position, taking Minecraft Education Edition (#2) and Festarola (#7) for instance. Minecraft Education was originally a sandbox game and later developed into an educational tool that supports students in exploring, building, and interacting in a dynamic virtual world to simulate real problem-solving scenarios. For example, students can design a “green city” that comprehensively considers energy use (such as solar panels), waste disposal, and transportation systems to simulate the sustainability challenges faced by real cities. Festarola, on the other hand, requires players to make strategic decisions around limited resources (such as time and budget) to achieve their goals.

In contrast, most interventions using gamification systems/tools are presented as a quiz/trivia (#5, 9, 10, and 11). These platforms (such as Kahoot! or custom-built quiz games) adopt a fast-paced answering mechanism, often supplemented by elements such as points, leaderboards, and instant feedback to enhance students’ participation. One of the specialized subcategories is the programming education platform (#6), and its core “quiz” activity involves using visual building blocks to solve programming challenges. The broader e-quiz/e-exercise system (#8) refers to comprehensive online platforms equipped with structured learning modules and integrated gamification-based progress tracking. It is worth noting that #9 covers both categories: it uses a serious game, My-Pet-Shop (RPG/simulations), to practice vocabulary application, and evaluates it with the help of a gamified quiz tool, My-Pet-Rush. Overall, the game genre analysis shows that serious games tend to promote learning through narrative immersion and complex task simulation, while the gamification system is mainly used to improve participation in practice and evaluation.

#### 4.3.2. Game Mechanics Related to Metacognitive Strategies

We analyzed the mechanics that explain how GBL can promote metacognitive learning (Table 11). Game mechanics refer to the specific rules embedded in GBL to foster students’ metacognitive strategies (Hunicke et al., 2004). The game mechanics in the studies are categorized by the phases (i.e., planning, monitoring, and evaluation) of metacognitive learning.

The analysis shows that the design mechanics supporting the planning strategy are diversified. Among them, the most common mechanism is explicit goal-setting (n = 8), that is, providing learners with clear, preset goals in the learning environment (for example, in study 1, learners choose from 20 goal modules). Closely related to it is the mission briefing/quest log (n = 7), which conveys, contextualizes, and continuously tracks learning objectives through narrative or task structure. In addition, participatory goal-setting (n = 1) is recognized, emphasizing that students work together to formulate behavior expectations (study 3). Other auxiliary mechanics include strategy selection menus for selecting approaches (n = 5), resource management for allocating limited resources (n = 4), sample analysis for providing initial guidance (n = 4), and pathfinding/map exploration for spatial planning (n = 2).

For the monitoring strategies, the mechanics of providing real-time perception and feedback are dominant. Among them, real-time feedback (n = 10) and progress bar/progress map (n = 9) are the most common, which can present performance indicators in real time and track learning progress visually. Other mechanics include timer/time pressure (n = 4) and AI companion or Non-Player Character (NPC) prompting (n = 4). Notably, one study used specific auditory cues/signals (the design of the “PAX Listen” sound in study 3). In addition, social reminder/peer reminder (n = 1), was also applied (study 3). Tools for recording and analyzing operations include in-game notepads/logs (n = 3) and system logic/code execution visualization (n = 3).

In terms of evaluation strategies, failure feedback and error-explanatory feedback are the most widely used mechanisms (n = 8). This design not only prompts students’ mistakes in time but also provides targeted explanations and guidance, significantly promoting learners’ reflection and adjustment of their cognitive processes. Secondly, end-of-level summaries (n = 6) and performance analytics (n = 5) provide students with review and result integration at the task-level and performance-data dimensions, respectively. In addition, the branching consequence mechanics (n = 2) support learners to evaluate the effectiveness of their own choices by presenting the differentiated results brought by different decision-making paths. It is worth noting that the mechanics of action playback have not been found in the existing literature.

#### 4.3.3. Methods for Measuring Metacognition in GBL

The frequency and distribution of the employed measurements are summarized in Table 12 to identify the most common methods for assessing metacognition in GBL studies. The findings show that self-reported tools, including questionnaires and scales, are the most commonly used methods, accounting for 54.55% of the reviewed studies. Second only to questionnaires, the integrated application of digital and behavioral trace data is also a recurring approach, appearing in four studies (36.36%). This method combination makes full use of the rich data in the digital game environment. The researchers firstly collected fine-grained and time-stamped in-game interaction records (digital trace), such as clicks, navigation paths and tool usage. These logs are then systematically coded and analyzed to construct higher-order behavior trace indicators reflecting the metacognitive process. Common derivative indicators include the frequency of plan adjustment, the sequence of help-seeking behavior and the text analysis of in-game reflection content. Other methods play a complementary role. A bit fewer than digital trace data, three studies (27.27%) employed direct observation to interpret the meaning of behaviors in classroom situations. Interviews (two studies) provide qualitative depth to capture students’ perceptions of metacognitive participation. Self-reflection analysis (two studies), mostly triggered by in-game prompts, provides a direct basis for students’ consciousness monitoring and evaluation process.

#### 4.3.4. Characteristics of GBL Interventions

Games or gaming elements are used as interventions to support student engagement and subject learning. Table 13 lists the games and gamified systems used in the reviewed literature, such as Festarola solving maths problems in study #7, and Oxford Achiever assisting English learning in study #8. The table also illustrates the organization of the intervention, briefly describing how students actually engaged with the games. These engagement forms are further categorized into three types: individual, group, or mixed. In the three studies (#2, 3, and 11), students collaborated to complete game tasks, whereas in the other six studies (#1, 4, 5, 6, 8, and 9), students worked independently rather than in groups. The remaining two studies (#7 and 10) employed a mixed approach, where students participated in both individual and collaborative gameplay across different sessions.

In addition, various game design elements are adopted to realize the above mechanics. For example, classic incentive components (such as points, badges, leaderboards and avatars) are integrated with data dashboards that provide detailed feedback (study 8). Similarly, the serious game Festarola constructs a learning environment (study 7) with both competition and feedback functions through avatars (player characters), collaborative design (such as team-based planning and purchasing tasks) and structured task flow of nested multidimensional scoring system.

In addition, duration of GBL interventions varies greatly, ranging from hours to one year (See Table 14). Intervention durations can be classified into four categories: short-term (≤4 weeks), medium-term (>4 weeks and ≤1 semester), long-term (>1 semester), and not specified. The majority of studies (45.45%, n = 5) fell into the medium-term category. For example, intervention specified in study 7 lasted eight weeks, with 60 min per session. Short-term interventions (≤4 weeks) were reported in three studies (27.27%), such as study 9 (two weeks, total 70 min). Long-term interventions (>1 semester) appeared in two studies (18.18%), including studies 1 and 10, both of which covered one academic year. Additionally, one study (#2) did not specify duration.

### 4.4. RQ4: Impact of GBL on Students’ Metacognitive Learning

This review identifies ten widely used GBL features in the reviewed studies: goal-setting, planning tools, immediate feedback, progress visualization, metacognitive strategy support, self-reflection, collaborative interaction, adaptive difficulty adjustment, emotional behavior support and gamification elements (See Table 15). Among them, immediate feedback, progress visualization, collaborative interaction and gamification elements have the highest frequency (each adopted in four studies), indicating that these features occupy a prominent position in empirical research and often associate with metacognitive monitoring, strategy adjustment and motivation improvement. The functions of goal-setting, plan support, explicit metacognitive teaching, adaptive difficulty and emotional support have also been verified in some studies, which together constitute a multidimensional scaffold system to promote metacognitive development. Overall, the GBL features effectively improve learners’ metacognitive awareness, strategy application and self-adjustment ability through structured guidance, real-time feedback, social interaction and emotional reinforcement.

## 5. Discussions

### 5.1. Key Findings

This scoping review synthesized recent empirical evidence on how game-based learning (GBL) supports metacognitive learning strategies among primary and junior middle school students. Based on the 11 reviewed studies, we find that GBL can support metacognitive learning when game-based environments are intentionally designed to scaffold planning, monitoring, and evaluation, with the strongest and most consistent support observed for monitoring-related processes through immediate feedback, progress visualization, and interactive task guidance. At the same time, the review shows that the evidence base remains methodologically uneven, developmentally imbalanced, and theoretically under-specified in important respects.

A first important finding is that the metacognitive value of GBL appears to depend less on whether an intervention is labeled a “serious game” or a “gamified system” and more on whether it embeds explicit regulatory scaffolds into gameplay. In the reviewed studies, planning was commonly supported through explicit goal-setting, mission briefings, strategy menus, and resource allocation structures, whereas monitoring was most frequently supported through real-time feedback and progress displays, and evaluation through explanatory error feedback, end-of-level summaries, and performance analytics. This pattern is theoretically coherent with cyclical SRL theory, in which effective learning requires learners to anticipate metacognitive activities. The present review, therefore, extends prior work that has broadly associated GBL with improved engagement and learning by showing more precisely which design mechanics are linked to specific metacognitive phases. This distinction is important because previous reviews have often treated game-based features as a relatively undifferentiated set of motivational affordances rather than as targeted supports for metacognitive regulation (Meng et al., 2024). Our review suggests that the educational value of GBL for metacognitive learning lies not in gamefulness per se, but in the alignment between mechanics and regulatory functions.

A second key finding concerns the especially prominent role of monitoring support. Among the identified mechanics, real-time feedback and progress visualization were the most frequently implemented, and they were also the most consistently associated with improved metacognitive awareness and strategy adjustment. This prominence is not surprising. Monitoring is the most externally observable component of metacognition and the easiest to operationalize within digital environments, where learner actions can be continuously tracked and mirrored back to the learner through dashboards, prompts, and status indicators. From the perspective of SRL theory, such features reduce the latency between action and consequence, thereby making the regulation process more visible and actionable for younger learners who may not yet spontaneously monitor their cognition in abstract ways. This interpretation is also consistent with work showing that computer-based prompts and metacognitive supports are most effective when they are timely, explicit, and tightly coupled to task performance rather than delivered as decontextualized advice (Guo, 2022; Dignath & Veenman, 2021). At the same time, the relative dominance of monitoring features reveals a gap in current design practice: planning and especially evaluation were scaffolded less comprehensively and often less deeply than monitoring. This imbalance may limit the extent to which GBL fosters fully cyclical SRL rather than short-term performance adjustment alone.

### 5.2. Implications

The review also highlights a developmental and contextual imbalance in the literature. Most included studies focused on primary school students, while junior middle school students were markedly underrepresented. This is a consequential gap rather than a simple sampling bias. Developmental research indicates that childhood and the transition into adolescence are critical periods for the formation, reorganization, and potential disruption of metacognitive self-regulation, especially as motivational profiles and learning demands shift across school stages (Katsantonis, 2024; Okada, 2021; Van Loon & Roebers, 2024). The predominance of primary-school evidence means that current conclusions about GBL-supported metacognitive learning are strongest for younger learners and should not be generalized uncritically to junior middle school settings, where motivational decline, increased academic pressure, and subject specialization may alter both the need for and responsiveness to metacognitive scaffolds. A similar caution applies to disciplinary distribution. The evidence was concentrated in STEM and language learning, with very limited representation from other educational domains. This concentration may reflect the relative ease with which task progression, correctness, and feedback can be formalized in STEM or language platforms, but it also narrows our understanding of how GBL might support metacognitive learning in less-structured domains that demand interpretation, argumentation, and perspective-taking. Accordingly, the field now needs designs that test whether the metacognitive benefits identified here are transferable across age groups and knowledge domains rather than domain-bound to highly structured digital tasks.

Another major contribution of this review is the identification of a methodological shift toward multimodal assessment, especially the growing use of digital trace and behavioral log data alongside questionnaires and scales. This is one of the most promising developments in the literature. Self-report measures remain highly prevalent because they are practical and scalable, but they are vulnerable to overestimation, limited introspective accuracy, and developmental constraints, particularly among younger learners (Craig et al., 2020). By contrast, digital trace data allow researchers to infer metacognitive processes from time-stamped interactions, such as planning-tool use, help-seeking sequences, navigation paths, and revision patterns. In principle, this offers a more process-sensitive and less reactive view of regulation in action. However, the present review also suggests that the field has not yet fully resolved the validity problem. Many studies collected trace data, but fewer clearly articulated how specific behavioral indicators mapped onto theoretically grounded constructs of planning, monitoring, or evaluation. In other words, the field is moving toward richer measurement, but not always toward stronger construct validity. This gap highlights a critical need for researchers to more explicitly define the relationships between observable actions and the underlying cognitive processes they aim to measure. Without this alignment, the validity of conclusions drawn from trace data remains uncertain, potentially limiting its usefulness in understanding metacognitive learning. Future research would therefore benefit from more explicit triangulation across self-report, trace data, reflective artifacts, and observational or verbal protocols.

The review further shows that theoretical grounding remains inconsistent across the literature. Although SRL-related models were the most common frameworks, nearly half of the studies did not clearly specify a guiding theory. This matters because theory is not simply an interpretive add-on; it determines how game features are selected, how metacognitive processes are defined, and how outcomes are measured. When GBL interventions are not anchored in a clear model of regulation, there is a risk that design features such as points, leaderboards, or rewards are used primarily to stimulate engagement without clarifying how they are expected to improve metacognitive functioning. This may help explain why the literature often reports positive effects on motivation, participation, or self-efficacy, but less consistently demonstrates how those effects translate into durable gains in planning, monitoring, or evaluation. Earlier reviews have similarly noted that the game-based learning field is theoretically heterogeneous, with uneven integration of educational psychology into design decisions (Krath et al., 2021; Wang & Huang, 2021). Our review sharpens this concern in the specific context of metacognitive learning: if the goal is to cultivate transferable metacognitive engagement rather than merely increase participation, then design must be explicitly theory-driven. In practice, this means integrating mechanics that correspond to SRL phases, sequencing supports so that learners progressively internalize them, and measuring outcomes in ways that distinguish metacognitive regulation from adjacent constructs such as motivation or behavioral engagement.

Several implications follow for educational practice and design. For teachers and designers, the evidence suggests that effective GBL for metacognitive development should combine clear goal structures, in-task monitoring supports, and post-task reflection opportunities rather than relying on isolated gamification elements. Immediate feedback and progress indicators are useful entry points, but they should be complemented by mechanisms that prompt learners to formulate plans before action and evaluate strategy effectiveness after action. For younger learners in particular, externalized supports may be necessary at first, but the ultimate design goal should be gradual internalization of regulatory routines. Collaborative structures may also be beneficial when they generate peer explanation, joint planning, and shared reflection, although the current evidence does not yet allow strong conclusions about when collaboration outperforms individual play. For researchers, the findings point to three priorities: stronger theoretical specification, more balanced attention to all three metacognitive phases, and more rigorous multimethod assessment. These priorities are especially important if the field intends to move beyond documenting short-term in-game effects toward demonstrating broader transfer to classroom learning.

### 5.3. Limitations and Future Research

This review also has limitations that should shape interpretation. As a scoping review, its purpose was to map the field rather than quantify pooled effect sizes, so the conclusions concern patterns of evidence rather than causal magnitude. The corpus was small, the intervention durations were highly heterogeneous, and some studies lacked detailed reporting of design logic or measurement procedures. Thus, this review does not allow for drawing generalizable and firm conclusions about effectiveness, but rather establishes a “snapshot” of the current state of research and points out existing gaps.

The restriction to English-language, peer-reviewed, open-access articles from 2015 to 2025 may also have excluded relevant work. In addition, because many studies combined metacognitive outcomes with broader constructs such as motivation, self-efficacy, or academic performance, the boundaries of what counted as metacognitive improvement were not always equally strict across the literature. Additionally, we did not conduct an assessment of methodological quality or risk of bias, which is typically conducted in systematic/meta-analysis reviews to evaluate the rigor of included studies. Thus, conclusions should be interpreted with caution. However, given that this is a scoping review, the primary aim is to map the available evidence, explore trends, and identify gaps in the literature rather than critically appraising study quality.

Nevertheless, these limitations do not weaken the main conclusion; rather, they clarify the field’s current maturity. The evidence is already sufficient to support the claim that GBL can promote metacognitive learning in primary and junior middle school education when its mechanics are intentionally aligned with regulatory processes. What remains insufficient is a more developmentally balanced, theoretically coherent, and methodologically robust body of evidence capable of explaining not only whether GBL works, but also for whom, under what design conditions, and to what degree transfer occurs beyond the game environment.

## 6. Conclusions

In conclusion, this scoping review demonstrates that game-based learning (GBL) has clear potential to support metacognitive learning, especially in terms of planning, monitoring, and evaluation, among primary and junior middle school students. Across the reviewed studies, the most effective support appeared in game environments that intentionally embedded metacognitive scaffolds, particularly explicit goal-setting, real-time feedback, progress visualization, and post-task reflection. These findings indicate that the contribution of GBL to metacognitive learning depends less on the mere presence of game elements and more on whether game mechanics are deliberately aligned with the cyclical processes of self-regulated learning. In addition, this review offers practical and conceptual guidance for both researchers and practitioners. For educators and designers, it highlights the importance of building GBL environments that scaffold learners’ planning before action, monitoring during action, and evaluation after action. For researchers, it underscores the need for stronger theory-driven design, more developmentally balanced samples, and more rigorous multimethod assessment of metacognitive processes. As GBL continues to develop, its educational value will depend on whether future work can move beyond engagement alone and more systematically demonstrate how game design can cultivate transferable metacognitive competence in school learning.

## Figures and Tables

**Table 1:** Keywords for literature retrieval.

Core Concepts	Keywords
Game-Based Learning	(“game-based learning” OR “serious games” OR “educational games” OR “digital game-based learning” OR “learning games” OR “game-based simulations”)
Metacognition & Self-Regulation	(“metacognition” OR “metacognitive” OR “self-regulated learning” OR “self-regulation” OR “self-monitoring” OR “self-evaluation” OR “learning strategies”)
Primary & Junior Middle School	(“elementary school” OR “primary school” OR “middle school” OR “junior high” OR “junior high school” OR “intermediate school” OR “elementary education” OR “primary education” OR “secondary education”)

**Table 2:** Inclusion and exclusion criteria.

Standards	Inclusion Criteria	Exclusion Criteria
Literature types	Research that uses qualitative or quantitative methods to collect empirical data	Non-empirical research articles (such as literature review, meta-analysis, comments, editorials, and theoretical articles)
Participants	Pupils or junior middle school students	Preschool children, senior high school students, college students, adults or learners in informal education environment.
Research forms	Include gaming activities	Teaching intervention without game elements
Results/Measurement	Includes detailed measurement results and analysis of students’ metacognitive strategies (such as planning, monitoring and evaluation)	Lacks rigorous data analysis and detailed results.

**Table 3:** Codes corresponding to RQs.

Research Questions	Coding
RQ1	Theoretical foundations for the design of GBL
RQ2	Game design types integrated in GBL for metacognitive learning, Game mechanics used in metacognitive strategies (planning, monitoring, and evaluation Specific serious games or gamified systems as intervention tools, Types of organization for intervention (individual, group, or hybrid), and duration of intervention
RQ3	Impact of GBL features on students’ metacognitive learning

**Table 4:** Sample sizes of the reviewed studies.

Sample Size	Study ID	Count (Percentage)
Fewer than 50	9, 11	2 (18.18%)
50–200	1, 4, 5, 6, 8	5 (45.45%)
Greater than 200	7, 10	2 (18.18%)
No specified	2, 3	2 (18.18%)

**Table 5:** Educational levels of the participants.

Students	Study ID	Count (Percentage)
Primary schools (Grade 1–6)	2, 3, 5, 6, 7, 8, 9, 10, 11	9 (81.8%)
Junior middle schools (Grade 7–9)	1, 4	2 (8.2%)

**Table 6:** Subject domains.

Domain	Domains (Study ID)	Count (Percentage)
STEM	Microbiology (1, 4); Informatics education (2); Mathematics (5, 7); Computational thinking (6)	6 (54.5%)
Language Learning	English vocabulary (9); English as a foreign language (10); English as a second language (8, 11)	4 (36.4%)
Other education domains	Special Education (3)	1 (9.1%)

**Table 7:** Research settings of the included studies.

Research Context	Study ID	Count (Percentage)
Physical classroom	2, 3, 4, 5, 6, 7, 9, 11	8 (72.73%)
Online environments	1, 8	2 (18.18%)
Mixed	10	1 (9.09%)

**Table 8:** Research methods used in the selected papers.

Research Context	Study ID	Count (Percentage)
Quantitative	1, 4, 5, 6, 9	5 (45.45%)
Qualitative	3	1 (9.1%)
Mixed	2, 7, 8, 10, 11	5 (45.45%)

**Table 9:** Theoretical frameworks used.

Theory	Study ID	Count
Self-Regulated Learning Theory	1, 4, 6, 9, 11	5
Self-Determination Theory	5	1
Other (Situated Learning Theory, Self-Regulated Learning Model, Distributed Practices)	9	1
Not specified	2, 3, 7, 8, 10	5

**Table 10:** Game types/genres used in the articles.

Fundamental for GBL	Game Types/Genres (Study ID)	Count (Percentage)
Serious Game	adventure/RPG (#1, 4, 9); simulations (sandbox, resource management) (2, 3, 7)	5 (45.5%)
Gamified System	quiz/trivia (5, 9, 10, 11); programming education platform (6); e-quiz/e-exercise system (8)	6 (54.5%)

**Table 11:** Game design mechanics utilized in metacognitive strategies phases.

Metacognitive Strategies	Game Design Mechanics	Study ID	Count
Planning	Explicit goal-setting	1, 2, 3, 4, 7, 8, 9, 11	8
	Mission briefing/quest log	1, 2, 3, 4, 7, 9, 11	7
	Strategy selection menus	1, 4, 5, 6, 9	5
	Resource management	1, 7, 9, 11	4
	Sample analysis	2, 3, 4, 5	4
	Pathfinding/Map exploration	7, 11	2
	Participatory goal-setting	3	1
Monitoring	Real-time feedback	2, 3, 4, 5, 6, 7, 8, 9, 10, 11	10
	Progress bars/maps	2, 3, 5, 6, 7, 8, 9, 10, 11	9
	Timers/time pressure	3, 7, 9, 10	4
	AI companion/NPC prompts	2, 5, 7, 8	4
	In-game notepads/logs	1, 2, 9	3
	Visualization of system logic/code execution	2, 6, 8	3
	Auditory cues	3	1
	Social prompts (peer reminders)	3	1
Evaluation	Feedback on failure/Explanatory feedback on errors	2, 3, 4, 5, 6, 7, 8, 9	8
	End-of-level summaries	2, 3, 5, 7, 9, 11	6
	Performance analytics	5, 7, 8, 9, 10	5
	Branching consequences	7, 9	2

**Table 12:** Methods for measuring metacognition in the articles.

Method (Count)	Description	Study ID
Questionnaires (n = 6)	Includes Likert scales, and open-ended questionnaire items.	2, 4, 5, 8, 9, 11
Digital Trace Data (n = 4)	The collection and analysis of digital backend logs (digital trace) to derive coded indicators of specific learning behaviors.	1, 6, 9, 10
Observations (n = 3)	Direct observation by researchers or teachers.	2, 10, 11
Interviews (n = 2)	Semi-structured or focus group interviews.	3, 8
Self-reflections (n = 2)	Analysis of students’ written or in-game reflection prompts.	7, 10
Other (n = 1)	Analysis of meeting minutes, researcher journals	10

**Table 13:** Type of organization for GBL interventions.

Type of Organization for Intervention	Studies	Count (Percentage)
Individual	1, 4, 5, 6, 8, 9	6 (54.55%)
Group	2, 3, 11	3 (27.27%)
Mixed	7, 10	2 (18.185%)

**Table 14:** GBL intervention duration.

Category	Duration Range	Study ID	Count (Percentage)
Short-term	≤4 weeks	4, 9, 11	3 (27.27%)
Medium-term	>4 weeks and ≤1 semester	3, 5, 6, 7, 8	5 (45.45%)
Long-term	>1 semester	1, 10	2 (18.18%)
Not specified	—	2	1 (9.09%)

**Table 15:** Impact of GBL features on metacognitive learning.

Feature	Impact	Study ID	Count
Goal-Setting	Enhances students’ awareness and ability to set learning objectives	2, 11	2
Planning Tools	Promotes goal-setting and planning, strengthens metacognitive planning ability	2, 7	2
Immediate Feedback	Facilitates self-monitoring and adjustment, enhances metacognitive awareness	1, 6, 10, 11	4
Progress Visualization	Strengthens goal-setting and progress monitoring in metacognition	1, 6, 8, 9	4
Metacognitive Strategy Support	Helps students identify and apply metacognitive strategies	1, 11	2
Self-reflection	Promotes students’ reflection on and evaluation of learning outcomes	4, 7, 11	3
Collaboration & Peer Interaction	Facilitates reflective and strategic adjustment in metacognitive processes	1, 6, 7, 8	4
Adaptive Difficulty Adjustment	Supports ability matching and sustained challenge, enhances self-efficacy	7, 8	2
Emotional & Behavioral Support	Enhances self-awareness and emotional regulation, promotes metacognitive control	3, 10	2
Gamification Elements (points, rewards, narrative, etc.)	Increases motivation, self-efficacy, and engagement, thereby supporting the use of metacognitive strategies	1, 6, 8, 11	4

## Data Availability

Dataset available on request from the authors.

## Abbreviations

GBL	Game-Based Learning
NPC	Non-Player Character
PRISMA	Preferred Reporting Items for Systematic Reviews and Meta-Analyses
RQs	Research Questions
RPG	Role-Playing Game
SDT	Self-Determination Theory
SRL	Self-Regulated Learning
STEM	Science, Technology, Engineering, and Mathematics

## Appendix A. List of the Reviewed Studies

**Table A1:** List of the reviewed articles.

Study ID	Authors, Year	Journal	Countries/Regions
1	(Goslen et al., 2024)	*Journal of Educational Psychology*	United States
2	(Riabko & Ihnatenko, 2025)	*Scientific Bulletin of Mukachevo State University. Series “Pedagogy and Psychology”*	Ukraine
3	(Jornevald et al., 2025)	*European Journal of Psychology and Educational Research*	Sweden
4	(Geden et al., 2021)	*International Journal of Artificial Intelligence in Education*	United States
5	(Leonardou et al., 2020)	*Information*	Greece
6	(C.-Y. Chen et al., 2023)	*Sustainability*	Taiwan, China
7	(Rodrigues et al., 2020)	*IEEE Computer Graphics and Applications*	Portugal
8	(X. Li et al., 2022)	*Sustainability*	China
9	(Z.-H. Chen & Shu-Yu, 2018)	*Journal of Educational Technology & Society*	Taiwan, China
10	(Zou, 2020)	*Journal of Computers in Education*	Hong Kong, China
11	(Noriega Cortes & Carreño-Bolivar, 2024)	*F1000Research*	Colombia
